# Increased engagement of the cognitive control network associated with music training in children during an fMRI Stroop task

**DOI:** 10.1371/journal.pone.0187254

**Published:** 2017-10-30

**Authors:** Matthew Sachs, Jonas Kaplan, Alissa Der Sarkissian, Assal Habibi

**Affiliations:** Brain and Creativity Institute, Dornsife College of Letters, Arts and Sciences, University of Southern California, Los Angeles, California, United States of America; Universiteit Gent, BELGIUM

## Abstract

Playing a musical instrument engages various sensorimotor processes and draws on cognitive capacities collectively termed executive functions. However, while music training is believed to associated with enhancements in certain cognitive and language abilities, studies that have explored the specific relationship between music and executive function have yielded conflicting results. As part of an ongoing longitudinal study, we investigated the effects of music training on executive function using fMRI and several behavioral tasks, including the Color-Word Stroop task. Children involved in ongoing music training (N = 14, mean age = 8.67) were compared with two groups of comparable general cognitive abilities and socioeconomic status, one involved in sports (“sports” group, N = 13, mean age = 8.85) and another not involved in music or sports (“control” group, N = 17, mean age = 9.05). During the Color-Word Stroop task, children with music training showed significantly greater bilateral activation in the pre-SMA/SMA, ACC, IFG, and insula in trials that required cognitive control compared to the control group, despite no differences in performance on behavioral measures of executive function. No significant differences in brain activation or in task performance were found between the music and sports groups. The results suggest that systematic extracurricular training, particularly music-based training, is associated with changes in the cognitive control network in the brain even in the absence of changes in behavioral performance.

## Introduction

It is well documented that music training can be associated with improvements in various cognitive abilities and brain functioning, particularly in the realm of auditory processing [1]. However, the extent to which learning to play an instrument promotes nonmusical skills is still debated [2]. Furthermore, the neural mechanisms that could account for such far-transfer effects to non-auditory skills have yet to be clarified. Some researchers have proposed that music training promotes the development of executive function, which, in turn, leads to the enhanced cognitive abilities [3]. Executive function is generally conceptualized as three interrelated cognitive processes, inhibition, working memory, and cognitive flexibility, that, together, allow for goal-directed behavior and cognitive control [4]. *Inhibition*, or *inhibitory control*, is the ability to resist and control one’s attention, behavior, and thoughts that may be habitual or impulsive, often in favor of more appropriate responses. *Working memory* is the ability to keep important and relevant information in mind while simultaneously performing another task. *Cognitive flexibility*, or *task switching*, is the ability to quickly and easily switch between different tasks, incorporating and adjusting to changing demands or new information. These cognitive capacities begin to develop in early childhood and continue to improve through adolescence [4].

Executive functions have been shown to robustly correlate with various metrics of health, achievement, wealth, and quality of life [5]. The development of cognitive control appears to be particularly predictive of health, success and wellbeing later in life [6]. Because of this strong association between the development of cognitive control and positive measures of human behavior, a large body of research has focused on investigating the ways in which such skills can be improved, particularly in early childhood. Such studies have shown that executive functions can be enhanced by consistent and intensive cognitive training programs in 7 to 12 year olds [7,8], video game training in older adults [9], as well as martial arts [10], mindfulness meditation [11,12], aerobic exercise [13], and sports training in children between the ages of 5 to 10 [14]. However, not all of these studies included an active control groups or collected both pre- and post-training measures and thus strong evidence for far-transfer effects associated with training remains limited.

Playing a musical instrument requires utilizing many of the same cognitive mechanisms that constitute executive functions. During a musical performance, a musician has to continually and seamlessly switch between reading notes, monitoring and adjusting necessary motor actions, and attending to new and competing streams of auditory information coming from other performers as well as one’s own playing [15]. Given the complexity of playing an instrument, it may not be surprising that studies have reported training-related changes in brain regions involved in both auditory and non-auditory cognitive processes in association with music training [16]. However, empirical investigations that have specifically tested the hypothesis that music training is associated with enhanced executive functions have reported mixed results and thus, such a link remains elusive.

It has been proposed that inhibitory control and conflict processing in particular might mediate the transfer of skills from training to cognitive abilities [3], yet the evidence for this model is weak given the quasi-experimental design of the majority of studies that have assessed the relationship between music training and cognitive control. Several studies have found evidence for enhanced performance on the Stroop task, a commonly-used task of cognitive control [17], associated with musical expertise. Professional musicians demonstrated faster reaction times on the Color-Word Stroop task than an age-matched group of amateur musicians [18] and young adults with musical experience demonstrated significantly faster reaction times than non-musicians on both the Simon arrow task and an auditory Stroop task [19]. Faster reaction times during the auditory Stroop task was also found with older professional musicians compared to non-musicians, though other measures of inhibitory control did not differ between groups [20]. Both the amount of music training and degree of musical ability have also been shown to correlate with better performance on auditory and visual Stroop tasks [15]. Furthermore, participants with musical expertise demonstrated better prediction skills when cues were presented aurally, visually, and cross-modally [21], suggesting that music training might influence one’s ability to predict future events based on previously presented stimuli, a skill believed to be a prerequisite for several cognitive functions and learning.

Additionally, several studies have reported a relationship between music training and performance on working memory tasks, in particular, verbal working memory [22,23]. On the other hand, a recent cross-sectional study failed to replicate these results, reporting no differences between adult musicians and matched controls on either a working memory task or a visual inhibition task [24]. Another cross-sectional study did find that musical ability was positively associated with performance on auditory and visual tests of working memory in adults, but it was not associated with performance during tasks that probe inhibition nor task-switching [25].

The relationship between music training and executive function is even less clear in children. In one study, musically trained 9–12 year olds scored higher on a measure of IQ and performed better on the Digit Span, a working memory task, than untrained children; however, none of the other executive function tasks were significantly different between the two groups and the association between IQ and music training was not mediated by performance on such tasks [2]. In a follow-up study, in which amount of music training was assessed continuously rather than as a binary, categorical variable, a significant positive relationship was found between years of music lessons and performance on tasks of executive function that assessed task switching, inhibitory control, and selective attention [26]. Corroborating these findings, a recent large-scale study reported that improvements from year to year on a visuo-spatial and verbal working memory task were predicted by the amount of time 6–25 year olds had spent practicing a musical instrument [27].

Several factors may account for the inconsistent findings, including a failure to consider potential confounds such as socio-economic status, length and amount of training, and age-related difference, as well as the lack of an active control group that also received training, but in a non-musical activity. In addition, due to the cross-sectional design implemented in the studies mentioned above, it cannot be concluded whether the improvements in executive functions, if present, were primarily the result of music training or if children with pre-existing enhanced cognitive abilities were drawn to music in the first place. Alternatively, both an interest in music and executive function could be promoted by some third, unaccounted-for variable. Longitudinal studies using music-based interventions have the potential to address this issue. Studies assessing executive functions before and after a period of music-based interventions have shown that music training is associated with enhancements in working memory [28,29], performance on an inhibition task [30], and on a test of attentional control [29]. A recent longitudinal study with children found that random assignment in an after-school music training program was associated with enhanced performance on several tasks of cognitive control and response inhibition [31]. However, an effect of music-based interventions on measures of executive function was not replicated in another large-scale study of children between the ages of 6–14 [32].

Neuroimaging may additionally help clarify the link between music training and executive functions by illuminating structural and functional differences in the brain that may not be captured by behavioral assessments alone. fMRI studies have revealed that the three components of executive function all engage a network of brain regions subserving cognitive control, including the dorsolateral prefrontal cortex (DLFPC), inferior frontal gyrus (IFG), anterior cingulate cortex (ACC), supplementary motor cortex (SMA) and pre-supplementary motor cortex (pre-SMA), precuneus, and insula [33]. Based on these results, several models have been developed to account for how these brain regions function together to enable successful cognitive control, highlighting the importance of the ACC specifically for such abilities. According to such models, the DLPFC is involved in regulating selective attention, the IFG enables rule representation and task-switching, the ACC serves as a conflict monitor, and the SMA/pre-SMA is required for selecting the appropriate, as well as inhibiting inappropriate, automatic motor actions [34]. The ACC also appears to be involved in a wide range of cognitive tasks that extend beyond cognitive control. Recent accounts have attempted to unify these extensive findings, arguing that the ACC might be involved in predicting the likelihood of an error [35] as well as the value of the possible future outcomes of exerting control, rather than monitoring conflict specifically [36].

Additionally, studies have shown that the magnitude of signal change within these regions is correlated with better performance on executive function tasks [37,38]. For example, the degree of engagement of the dorsal ACC during a cognitive control task was found to be correlated with academic achievement in medical students, suggesting a possible neural mechanism by which the development of cognitive control can become associated with positive life outcomes [39]. Conversely, hypoactivity in the DLPFC, IFG, ACC, SMA and pre-SMA has been observed in clinical populations with known impairments in cognitive control, such as patients with schizophrenia [40], attention-deficit hyperactivity disorder (ADHD) [41], and drug addictions [42], though the exact relationship between degree of activation in these regions and cognitive control performance is still a matter of debate [43]. Overall, previous neuroimaging findings suggest that greater recruitment in this network of brain regions is associated with enhanced cognitive control [33] and that greater activity in the ACC in particular may be associated with successful conflict processing and resolution [44].

Training-related changes within these regions have also been reported in adults after several weeks of cognitive training on a cognitive control task [45], in 7–11 year-old children after several months of an exercise program [46], and between 9–10 year-old children with high versus low aerobic fitness [47,48]. To our knowledge, however, while various studies have reported an association of early music training with specific changes in brain structure and functioning [49,50], few studies have investigated the neural differences between musicians and non-musicians during tasks that probe cognitive control specifically. One such study found that child musicians (9–12 years-old) displayed increased activity bilaterally in the SMA and the IFG during tasks that required rule representation and cognitive flexibility as compared to a control group despite no significant group differences in measures of in-scanner performance of the task [51]. Another neuroimaging study assessed working memory in adults and found that adult musicians performed better and showed heightened activity in the precentral gyrus, SMA, IFG, insula, and ACC during a working memory task that used musical chords [52]. Unfortunately, neither of these studies included an active control group and thus it is unclear if music training uniquely impacts executive functions or, if instead, the reported enhancements in executive functions could be produced by any rigorous and socially engaging type of training.

The goal of the current study was to clarify the connections between music training and executive function in developing children using neuroimaging. As part of a larger, 5-year longitudinal study, we evaluated performance on various measures of executive function and activity in brain regions involved in cognitive control in children engaged in a well-known, standardized music curriculum primarily involving group-based instruction on string instruments (violin and viola). We compared these children (music group) to two matched control groups: one involved in sports training for the same amount of time (sports group), and one not involved in systematic training in either music or sports (control group). The inclusion of the sports group allowed us to assess whether any observed differences are uniquely associated with music training or are associated with involvement in an extra-curricular activity more generally.

By comparing behavioral performance on tasks of working memory, inhibitory control, and cognitive flexibility, as well as neural responses during a task of cognitive control between these three groups, we aimed to determine the unique contributions of music training on the development of executive functions. We chose to use an fMRI Color-Word Stroop task because it is well validated in developmental neuroimaging studies of conflict processing [53] and because of the ambiguity in the literature regarding the nature of the relationship between cognitive control and music training.

We hypothesized that (1) children who have received systematic music training would perform significantly better on behavioral measures of executive function as compared to the control group with no systematic training in either music or sports; (2) during the Color-Word Stroop task while in the MRI scanner, musically-trained children would show significantly greater recruitment of brain regions consistently reported to be involved in cognitive control, including the IFG, SMA, pre-SMA, ACC, and insula, as compared to the control group. Additionally, we compared behavioral measures of executive function and BOLD signal during the Stroop task between children with music training and children with sports training to evaluate whether the specific auditory, sensorimotor and cognitive skills that are acquired when learning music are associated with additional enhancements above and beyond those that are accounted for by any type of systematic training.

## Materials and methods

### Participants

Participants were part of an ongoing longitudinal study addressing the impact of music training on cognitive, socioemotional, and neural development [54] and were recruited from public elementary schools and community music and sports programs in the greater Los Angeles area. All children were initially recruited at the age of 6, before any training began, and various behavioral measures of cognitive functioning were evaluated at baseline, i.e. before the onset of any systematic training. There were no differences between the groups in age, gender, socioeconomic status, and cognitive abilities at baseline assessment [54].

After two years, participants completed the first functional MRI scan. The distribution of participants in this study was as follows: Eighteen children (13 boys and 5 girls, 1 left-handed, mean age at the time of scan = 8.69 years, SD = 0.44, age range = 8.08–9.42) had been participating in the Youth Orchestra of Los Angeles at Heart of Los Angeles program (hereafter called “music group”). Eighteen children (8 boys and 10 girls, 1 left-handed, mean age at the time of scan = 8.94 years, SD = 0.58, age range = 8.00–10.08) had been training in a community- based soccer or swimming programs and have not been engaged in any regular musical training; these formed the first control group (hereafter called “sports group”). Twenty children (12 boys and 8 girls, 2 left-handed, mean age at the time of scan = 9.05 years, SD = 0.48, age range = 8.33–9.92) were recruited from public schools in the same area of Los Angeles provided they were not involved in any systematic, regular after-school training programs and formed the second control group (hereafter called “control group”). At each year of the study, children and their parents were interviewed and asked to report any activities that they had begun to participate in outside of school. While some of the control participants did begin sports programs in subsequent years after the start of the study (a risk inherent to any developmental longitudinal design), on average, the intensity, duration, and regularity of the training programs in the control group after two years was still significantly less than the children in the sports group.

All three cohorts came from equally under-privileged minority, primarily Latino, communities of downtown Los Angeles. All children were raised in bilingual households, spoke fluent English and attended English speaking schools. Exclusion criteria included any history of psychiatric or neurologic disorders. At the time of initial recruitment, as well as at each follow-up assessment, participants were screened through an extensive parental interview to ensure that they had not developed any condition diagnosed as a developmental or neurological disorder. Clinical assessments were not used for screening.

### Description of music and sports after-school programs

#### Music training program: El Sistema & Youth Orchestra of Los Angeles

Participants in the music group took part in an El Sistema-inspired program called the Youth Orchestra of Los Angeles (YOLA), which offers free music training, five days a week, to children from underserved areas of Los Angeles. Acknowledged worldwide as the “most significant example of collective music education” [55], El Sistema is claimed by many to be an important mechanism of social change through music [55,56]. Since its inception in 1975, thousands of children, most from underserved communities, have gone through this publicly funded program in Venezuela. Through intensive, collective music learning experiences, El Sistema aims to promote inclusion and combat poverty by empowering at-risk children and youth, and providing them with high quality music learning experiences [57]. Aligned with the central tenets of El Sistema, YOLA emphasizes ensemble practice and group performances. To join the program, children were selected by lottery, (maximum of 20 per year), from a list of interested families. Once selected, the children were provided with a string instrument, either a violin or viola, which they can take home. The musical curriculum consists of 7 weekly hours of music learning, divided into string instruments, choir, Orff, and musicianship (ear training and theory skills). Practice outside of the program is left at the discretion of students and their parents. Importantly, none of the participants in the music group participated in regularly-scheduled, after-school, sports programs.

#### Sports training program

Participants in the sports group took part in one of the two following community-based sports programs, free of charge, that serve neighborhoods of downtown Los Angeles: (1) a soccer program which offers soccer training 3 times a week with an additional game each weekend for children aged 6 and older (2) a swimming program which offers free swim instruction 2 times a week to school-age children with an additional recreational swim session each weekend. Each session lasts approximately 1 hour. Participants in both sports programs enrolled voluntarily in their respective programs and both programs were taught by trained coaches. All students who signed up for the sports program were admitted provided they met low socio-economic criteria of the program. Importantly, none of the participants in the sports group participated in any regularly-scheduled, music-based, after-school training programs.

We selected sports training as a comparison to music because, like music training, it requires investment of time and effort, self-discipline and fosters social engagement. These aspects may, in themselves, promote social, cognitive and neural development. Including such a comparison group helps to specifically identify the beneficial effect of music training on the measures we proposed.

### Socio-economic status (SES)

Parents indicated their highest level of education and annual household income on a questionnaire. Responses to education level were scored on a 5-point scale: (1) Elementary/Middle school; (2) High school; (3) College education; (4) Master's degree (MA, MS, MBA); (5) Professional degree (PhD, MD, JD). Responses to annual household income were scored on a 5-point scale: (0) < $ 10,000 (1) $10,000 − $19,999 (2) $20,000–29,999 (3) $30,000–39,999 (4) $40,000–49,999 (5) > $50,000. Parents provided income level and education level at each year of the longitudinal study. For education, the higher of the two reported levels (one for each parent) was used for analysis. Six parents chose not to indicate their income and 6 parents chose not to indicate their education level at the time of the functional scan. For these participants, the income level or education level reported from previous years was used for subsequent analysis.

### Experimental procedure

Study protocols were approved by the University of Southern California Institutional Review Board. Informed consent for participation in the study was obtained in writing, from the parents/guardians in the preferred language, on behalf of the child participants and verbal assent, during each yearly visit, was obtained from all children individually. Either the guardians or the children could end their participation at any time. Parents/guardians received monetary compensation ($15 per hour) for their child’s participation and children were awarded small prizes (e.g. toys or stickers). Transportation, e.g. taxi vouchers or pre-paid public transportation tickets were provided for the participants if necessary. All children were tested individually at our laboratory at the Brain and Creativity Institute at the University of Southern California.

### Behavioral assessments

Children participated in the study on two separate days. On the first visit, participants completed the MRI, which included the fMRI Color-Word Stroop task, and the parents of participants completed a survey that assessed income and education level as well as their child’s background, typical schedule, and involvement in extra-curricular activities. On the second visit, children completed the behavioral battery (see below), which included the behavioral Color-Word Stroop task outside of the scanner. A description of each task is presented below.

#### Wechsler abbreviated scale of intelligence (WASI-II)

The WASI-II for children 6 years and older [58] was used to assess levels of general intelligence. Subtests included the Block Design, Vocabulary, Matrix Reasoning, and Similarities. In addition, children completed the Memory for Digit Spans (forward and backward) from Wechsler Intelligence Scale for Children. In the forward condition, the child listened to a sequence of numbers spoken by the experimenter and was instructed to verbally repeat the sequence in the correct order. In the backward condition, the child again listened to a sequence of numbers and was instructed to repeat them in reverse order. In both conditions, the length of the sequence of numbers increased with correct responses until two trials with the same length are answered incorrectly.

#### WASI and Digit Span analysis

An overall FSIQ score (M = 100, SD = 15) was calculated based on responses from all four subtests of the WASI-II. In addition, a separate Performance IQ (PIQ) was calculated based on only the Block Design and Matrix Reasoning subtests and Verbal IQ (VIQ) was calculated from the Similarities and Vocabulary subtests. These scores measured fluid and crystallized intelligence, respectively. Standardized T scores (M = 50, SD = 10) were also provided for each of the four subtests. Each score was based on norms from a large sample of children living in the US, calibrated separately based on age in three-month increments.

For the Digit Span, two scores were calculated, one for the forward condition and one for the reverse condition, which reflected the total number of correctly answered trials in each condition.

#### Behavioral Color-Word Stroop task

Participants performed the Color-Word Stroop task outside the fMRI scanner as a measure of reaction time and accuracy [59]. The behavioral task was designed to match the fMRI-compatible version as closely as possible. Children were instructed to name, aloud, the color of the ink of the presented word and to ignore the actual written word as quickly and accurately as possible. Participants were also instructed to press the space bar at the same time that they spoke their response. Vocalizations of the response was used over a manual, button-press to avoid additional confounding difficulties associated with mapping color names to finger responses [60]. The participants completed six separate blocks of the task, where each block consisted of 12 trials of either all congruent trials, in which the color of the word matched the written word, or all incongruent trials, in which the color of the word did not match the written word. Each trial was presented for 1700ms. The task always started with a congruent block. Before the task began, a training session was administered in which the child responded to 5 incongruent trials and 5 congruent trials. The child was unable to advance to the next question until they responded correctly. Each of the 4 ink colors appeared exactly three times in each block, but there were no restrictions on the number of times the written color was presented provided it was not the same as the color of the ink in the incongruent condition. Trials within blocks were randomly permutated to create two unique randomizations ("A" and "B"). Participants completed either permutation A or B. Stimuli were presented on a MacBook laptop computer using Matlab (Mathworks, Natick, MA, USA) and the Psychophysics Toolbox extension [61,62].

#### Behavioral Color-Word Stroop task analysis

For the Color-Word Stroop task, both response time and accuracy were averaged across all blocks of one condition (incongruent or congruent). Response time for each trial was recorded in Matlab when the participant pressed the space bar. Any trial in which the participant did not audibly vocalize the correct color of the ink was marked as incorrect by the experimenter. Any trial in which the participants failed to respond within the allotted time window (1700ms) was additionally considered an incorrect response. Only trials in which the participants responded correctly were included in the measure of average response time. Within each condition, any trial in which the response time was above two standard deviations from the mean of the individual was removed from subsequent analysis. The first trial of each block was removed in subsequent analysis to allow the participants to adjust to the task. A 2 x 3 between-within ANOVA was then conducted in R (https://www.r-project.org/) to evaluate the effect of condition (incongruent vs. congruent) and group (music, sport, control), as well as the interaction, on both response time and accuracy.

#### Hearts and Flowers task

**Fig 1:**
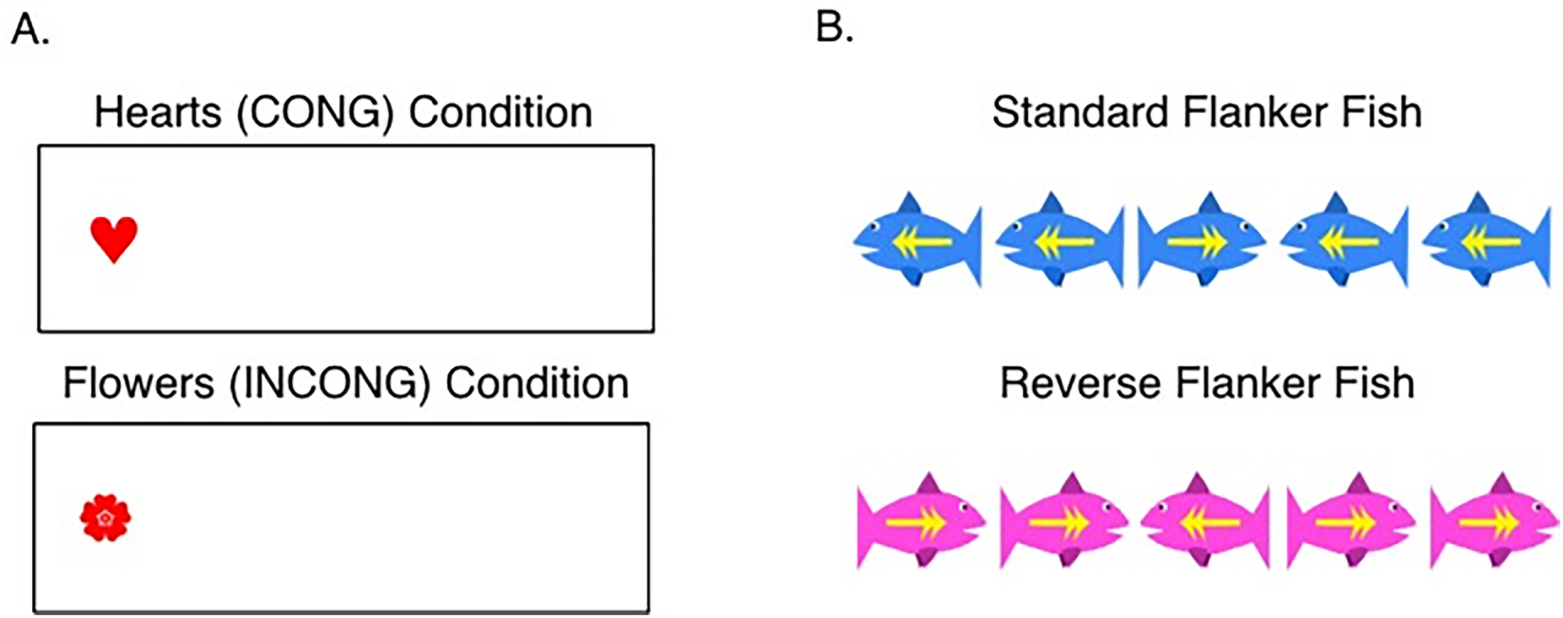
Hearts and Flowers and Flanker Fish tasks. (A) An example of a congruent, heart trial is on the top left, in which the child had to respond with the arrow button that was on the same side of the image, and an example of an incongruent, flower trial is on the bottom left, in which the child had to respond with the arrow button on the opposite side of the image. (B) An example of a trial in the standard condition of the Flanker Fish task is on the top right, in which the child had to respond with the arrow button corresponding to the direction of the middle fish, and an example of a trial in the reverse condition is on the bottom right, in which the child had to press the arrow button corresponding to the direction of the outside fish.

Outside the scanner, participants also completed the “Hearts and Flowers” task, which tests working memory, response inhibition, and task switching/cognitive flexibility [63,64]. The task required participants to press a response button that is either on the same side (congruent) or opposite side (incongruent) of an image. When children saw an image of a heart, they were instructed to press the corresponding directional arrow button on a keyboard. When children saw an image of a flower, they were instructed to press the opposite directional arrow button on the keyboard (see Fig 1A). The task consisted of three separate levels that contain only congruent trials (12 trials total), only incongruent trials (12 trials total), and mixed congruent and incongruent trials respectively (33 trials total). For each trial, participants had 750ms to respond. Images were presented on a MacBook laptop computer. Before both the congruent and incongruent trial blocks, a training period was administered which consisted of 5 trials. The child was unable to advance to the next question until they responded correctly.

#### Hearts and Flowers task analysis

For the Hearts and Flowers task, both response time and accuracy were recorded for each trial. The first trial of each block was removed. Within each condition for each subject, trials were considered incorrect (and subsequently not included in the response time calculations) if the participant did not respond within the allotted amount of time (750ms). Additionally, any trial in which the participant responded faster than 250ms was assumed to be too fast to indicate a reasonable respond and was also not included in response time calculations. Any participant who failed to accurately respond to at least 50% of the congruent trials was removed from the analyses. Ten participants were removed for this reason. In addition, data from 2 participants in the music group were not recorded correctly, which left a total of 13 participants in the control group, 9 in the music group, and 10 in the sports group for the final Hearts and Flowers task analysis.

Analysis focused on response time and accuracy during the mixed condition in which incongruent and congruent trials were intermixed, as this condition requires utilizing several components of executive function. A 2 x 3 between-within ANOVA was conducted for both response time and accuracy to evaluate group by condition interactions.

#### Flanker Fish task

Participants also completed a child-friendly version of the Flanker task called the “Flanker Fish” task [65] which assesses selective attention, task switching, working memory, and inhibitory control. Children were presented with a series of seven fish in a row and were required to press the response button that corresponds to either the direction that the middle fish is facing (standard condition) or the direction that the outside fish are facing (reverse condition). Which task was required was cued based on the color of the fish, with blue fish corresponding to the standard condition and pink fish corresponding to the reverse condition. In some of the trials, all the fish were facing the same direction (congruent condition) and in some of the trials, the outside fish were facing the opposite direction of the middle fish (incongruent condition, see Fig 1B). The task also consisted of three separate levels: a standard condition (17 trials total), reverse condition (17 trials total), and mixed condition (45 trials total) in which both standard and reverse trials were presented together. Each trial lasted for 1500ms or until the moment the child presses a response key, whichever came first. Before both the standard and reverse levels, a training period was administered which consisted of 5 trials. The child was unable to advance to the next question until they responded correctly.

#### Flanker Fish task analysis

Participants and specific trials were removed from analysis based on the same criteria as in the Hearts and Flowers task. Four participants, all from the sports group, incorrectly answered more than 50% of the congruent trials within the mixed flanker condition and were therefore removed from subsequent analysis. A 2x3 between-within ANOVA was conducted separately for response time and accuracy within each condition (standard, reverse, and mixed) to evaluate the interaction of group by condition.

### Neuroimaging assessment

#### fMRI Color-Word Stroop task

The children performed a modified version of the Color-Word Stroop task designed to be used inside the fMRI scanner [66]. During each trial, participants were presented with a word on a black screen written in one of four colors (red, yellow, green, or blue) and were instructed to subvocalize the color of the stimulus, regardless of the meaning of the written word, and simultaneously press an arbitrary button on a button box. Subvocalization, in which participants say the color of the ink in their heads rather than out loud, is a commonly used approach in developmental neuroimaging studies of cognitive control [53,67] because it helps minimize movement-related artifacts. On the other hand, the technique makes it inherently difficult to ensure that the participant is correctly completing the task. Therefore, extensive pre-scanning training was conducted and in-scanner response times between the conditions were evaluated to have on objective measure of whether the participant was indeed performing the task. Prior to scanning, participants practiced the task in a lab testing room. First, the child was presented with 3 blocks (1 congruent, 2 incongruent with 4 trials in each) and was instructed to say the color of the written word aloud. Second, the child was presented with 1 incongruent block (4 trials total) and was instructed to say the color of the word in his or her head and simultaneously press a button.

**Fig 2:**
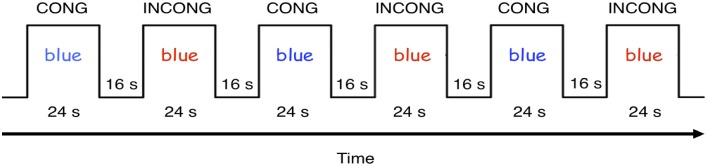
Color-Word Stroop task fMRI paradigm. Example of a single scanning session of the fMRI Color-Word Stroop task. Each run contained six blocks (three congruent, three incongruent). Each block contained 12 trials (1700ms per trial with a 300ms interval between). Each block was followed by a 16 second rest period for a total scan time of 240s per run.

During scanning, children completed two functional runs, each containing six blocks. Three blocks consisted of only congruent trials, in which the color of the stimulus and the text of the word matched, and three blocks consisted of only incongruent trials, in which the color of the stimulus and the text of the word did not match (Fig 2). As in the behavioral Stroop task, trials within blocks were pseudorandomized so that each block contained exactly three stimuli of every color. Randomization of trial orders was performed for each participant individually so that each participant saw a different order of stimuli. The blocks consisted of 12 trials, each presented for 1700ms with a 300ms interval between each trial (excluding the last trial in each block). The participants were instructed to respond as quickly and as accurately as possible. Each block was followed by a 16 second rest period (excluding the sixth and final block) for a total scan time of 240 seconds (120 TRs). Response time per condition was averaged for each participant and a between-within ANOVA was conducted to determine if response time was significantly different between conditions and between groups.

#### Imaging protocol

As part of the longitudinal battery, all children underwent anatomical, diffusion weighted, and functional MR imaging of their brain. Data collection parameters for the structural and functional scans only are described here.

We designed a child-friendly protocol that included a training session prior to the actual scanning session. Children learned about the scanner by watching a video and became acquainted with a scanning session in a mock scanner by listening to the different types of sound made by the scanner. They also practiced staying still during the mock session. During the actual scanning session, if children wished, one of the investigators remained in the scanner room and held their hand. After the scanning session, children were shown an actual image of their brain on the computer. Children watched a movie of their choice during the anatomical scan to assist them with staying still.

High-resolution T1-weighted structural MRI images were acquired using an MPRAGE sequence on a 3T MAGNETOM Prisma System equipped with a 20-channel head coil, with the following parameters: 1 mm × 1 mm × 1 mm resolution over a 256 mm × 256 mm × 256 mm FOV; TI/TE/TR = 850/32.05/2300ms; flip angle = 8°; GRAPPA acceleration factor *R* = 2.

Functional images were obtained using a gradient echo, echo-planar, T2*-weighted pulse sequence (TR = 2000 ms, one shot per repetition, TE = 25 ms, flip angle = 90°, 64 x 64 in-plane resolution). Forty-one slices covering the entire brain were acquired with a voxel resolution of 3 mm x 3mm x 3mm. One hundred sixty-five volumes were collected during each run of the fMRI Color Stroop task.

#### fMRI data analysis

Data were analyzed using FSL (FMRIB’s Software Library; http://fsl.fmrib.ox.ac.uk/fsl/fslwiki/). Pre-statistical processing included skull stripping (BET), motion correction (MCFLIRT), slice-timing correction, spatial smoothing (5.0 mm FWHM Gaussian kernel), and high pass temporal filtering (140 seconds). To further minimize effects of head motion, motion scrubbing was conducted for each functional run separately, using root-mean-squares intensity differences (dvars) as the metric of determining which slices should be regressed out in the GLM analysis [68]. Any slice in which the dvars exceeded the box-plot cutoff (values greater than 75^th^ percentile + 1.5*inter-quartile range) were included in a confound matrix that was subsequently added to the GLM model.

The two functional runs for each child were combined using a fixed effects analysis. Images were registered to a high-resolution structural and standard space using FLIRT with 12 DOF and a 2-mm MNI template. We modeled the task with a regressor for each of the two conditions (incongruent, congruent) using a boxcar convolved with a double-gamma hemodynamic response function. A general linear model was applied to contrast the BOLD signal between the two conditions of the task. Sessions level models were then combined into a higher level, mixed-effects analysis where independent two-sample t-tests were employed to examine differences in brain activation during these contrasts between the three groups. Given the developmental nature of this study, for all group-level analyses, age at the time of scan was included in the model as a covariate of non-interest. In a separate analysis, differences in response time between the two conditions of the behavioral Stroop task were also included as a covariate of interest. For both models, statistical inference was completed using Z images and FSL’s cluster thresholding, using a cutoff of Z > 2.3, and cluster size probability of p = 0.05. Unthresholded statistical maps were uploaded to NeuroVault.org database and are available at http://neurovault.org/collections/2396/.

Because of the known roles of the pre-SMA/SMA, ACC, and IFG in cognitive control [34], activation of these regions during the Stroop task was further investigated in a region of interest (ROI) analysis. Three spheres with radii of 8 voxels were drawn with the center coordinates located at the peak voxel (-2, 2, 58) within the pre-SMA/SMA, left IFG (-46,30,20), and right ACC (6, 16, 32) based on the significant clusters found in the group-level all subject, incongruent—congruent contrast. Percent signal change in the three ROIs was calculated for all participants from beta values using Featquery in FSL. The values were then correlated with averaged response time and accuracy during the incongruent conditions of the behavioral Stroop task conducted outside of the scanner and corrected for multiple comparisons. Because the neuroimaging data is being correlated with data that was collected outside of the scanner on a separate day, the two measures are independent from one other and thus, this analysis does not constitute “double-dipping” [69].

## Results

### Demographics

From the original 56 participants who completed the fMRI Stroop task, 12 subjects were removed (3 controls, 4 music, 5 sports) due to excessive motion (greater than 3mm mean displacement in either functional run), failure to correctly complete the task, or hardware malfunction, leaving a total of 44 remaining participants: 14 children (6 girls and 8 boys, 1 left handed, mean age at time of scan = 8.67 yrs., SD = 0.42, age range = 8.08–9.42) in the music group, 13 children (8 girls and 5 boys, 0 left handed, mean age at time of scan = 8.85 yrs., SD = 0.62, age range = 8.00–10.08) in the sports group, and 17 children (6 girls and 11 boys, 2 left handed, mean age at time of scan = 9.05 yrs., SD = 0.44, range = 8.33–9.92) in the control group.

Analysis revealed no significant differences in sex (χ^2^ (2, *N* = 44) = 2.10, p > 0.05, ϕ = 0.22), age (F(2, 41) = 2.09, p > 0.05, η^2^ = 0.09), socio-economic status (*Income*: F(2,41) = 1.51, p > 0.05, η^2^ = 0.07; *Education*: F(2,35) = 0.82, p > 0.05, η^2^ = 0.04), IQ (F(2, 41) = 2.23, p > 0.05, η^2^ = 0.10), or digit span (*Forward*: F(2,41) = 0.87, p > 0.05, η^2^ = 0.04; *Backward*: F(2,41) = 1.11, p > 0.05, η^2^ = 0.05) between the three groups and therefore these factors were not included in the subsequent analyses on behavioral performance. Age was included as a covariate for the fMRI analyses only. All demographic information is presented in Table 1.

**Table 1:** Participant characteristics.

	Control (n = 17)	Music (n = 14)	Sports (n = 13)
*Age*	9.05	8.66	8.84
*Gender*	11 M, 6 F	8 M, 6F	5 M, 8 F
*Handedness*	2 LH	1 LH	0 LH
*Income*	1.47 (1.23)	2.21 (1.48)	1.69 (0.75)
*Education*	2.40 (0.74)	2.77 (0.93)	2.60 (0.52)
*IQ*	98.59 (12.92)	105.29 (13.41)	95.77 (9.25)
*Digit Span Forward*	7.35 (1.5)	7.29 (1.44)	8.08 (2.25)
*Digit Span Backward*	4.71 (1.45)	4 (1.52)	4.54 (0.97)

### Executive functioning behavioral results

#### Behavioral Color-Word Stroop task

The between-within ANOVA revealed a significant main effect of condition on accuracy (F(1, 41) = 37.78, p < 0.001, η^2^ = 0.32). Average accuracy during the incongruent trials was significantly lower than accuracy during the congruent trials for all three groups [*control*: t(16) = 2.94, p < 0.05; *music*: t(13) = 5.13, p < 0.001; *sports*: t(12) = 5.24, p < 0.001]. There was no significant interaction between group and condition (F(2,41) = 0.29, p > 0.05, η^2^ = 0.01) or main effect of group (F(2,41) = 1.58, p > 0.05, η^2^ = 0.04).

A significant main effect of condition was also found for response time (F(1, 41) = 199.85, p < 0.001, η^2^ = 0.48). Response times during the incongruent trials were significantly longer than during the congruent trials for all three groups [*control*: t(16) = -9.41, p < 0.001; *music*: t(13) = -9.51, p < 0.001; *sports*: t(12) = -5.91, p < .001]. There was no significant interaction between group and condition (F(2,41) = 1.25, p > 0.05, η^2^ = 0.01) nor a significant main effect of group (F(2,41) = 1.18, p > 0.05, η^2^ = 0.04). The results are presented in Table 2.

**Table 2:** Mean accuracy and response time during both the behavioral and fMRI Color-Word matching Stroop task.

	Control		Music		Sports		Total	
	*Acc*.	*RT*	*Acc*.	*RT*	*Acc*.	*RT*	*Acc*.	*RT*
*I*	0.90 (0.10)	909 (188)	0.92 (0.06)	1008 (90)	0.93 (0.04)	933 (163)	0.91 (0.07)	947 (157)
*C*	0.98 (0.04)	650 (125)	0.99 (0.01)	695 (152)	0.99 (0.01)	693 (124)	0.99 (0.03)	677 (133)
*I > C*	-0.08 (0.11)	259 (113)	-0.08 (0.06)	313 (123)	-0.06 (0.04)	239 (146)	-0.08 (0.08)	259 (113)
*fMRI I > C*	--	115 (177)	--	189 (128)	--	128 (107)	--	144 (137)

Response time measured in ms and accuracy measured in percent correct. I = incongruent condition, C = congruent condition. SD in parentheses.

#### Hearts and Flowers task

Accuracy during the hearts (congruent) condition was significantly higher than the accuracy during the flowers (incongruent) condition (*hearts*: M = 0.98, S.D = 0.04; *flowers*: M = 0.86, S.D. = 0.12, t(41) = 6.08, p < 0.001). Response time was also significantly faster in the heart (congruent) condition, than the flower (incongruent) condition (*hearts*: M = 408.97ms, S.D = 66.58ms; *flowers*: M = 550.24ms, S.D. = 89.95ms, t(41) = -11.50, p < 0.001).

In the mixed condition, neither accuracy nor response time were significantly different between hearts and flowers trials (*accuracy*: F(1,29) = 0.59, p > 0.05, η^2^ = 0.01; *response time*: F(1,29) = 2.53, p > 0.05, η^2^ = 0.01) nor was there a significant interaction effect with group (*accuracy*: F(2,29) = 0.35, p > 0.05, η^2^ = 0.01; *response time*: F(2,29) = 0.40, p > 0.05, η^2^ = 0.002). The results are presented in Table 3.

**Table 3:** Mean accuracy and response time during the three conditions of the Hearts and Flowers task.

	Controls		Music		Sports		Total	
	*Acc*.	*RT*	*Acc*.	*RT*	*Acc*.	*RT*	*Acc*.	*RT*
*Hearts Condition*	0.96 (0.06)	419.82 (81.8)	0.98 (0.04)	396.38 (48.49)	0.99 (0.03)	406.41 (61.62)	0.98 (0.04)	408.70
*Flowers Condition*	0.84 (0.11)	544.22 (82.29)	0.91 (0.09)	531.43 (100.61)	0.85 (0.15)	575.48 (90.77)	0.86	550.24
*Mixed Condition*	0.08 (0.21)	30.10 (73.20)	0.10 (0.22)	29.52 (67.55)	0.03 (0.18)	24.34 (70.59)	0.07 (0.2)	28.07 (69.07)

Response time measured in ms and accuracy measured in percent correct. I = incongruent, C = congruent. SD in parentheses.

#### Flanker Fish task

In the Standard Flanker Fish, a marginally significant main effect of condition was found for accuracy (F(1,34) = 3.22, p = 0.08, η^2^ = 0.04) and a significant a main effect of condition was found for response time (F(1,34) = 7.58, p < 0.05, η^2^ = 0.06). In general, participants were more accurate (marginal) and faster during the congruent conditions within the Standard Flanker task (Table 4). No group by condition interaction was found for either accuracy (F(2,34) = 0.41, p > 0.05, η^2^ = 0.012) or response time F(2, 34) = 0.56, p > 0.05, η^2^ = 0.008).

**Table 4:** Mean accuracy and response time during the three conditions of the Flanker Fish task.

	Controls		Music		Sports		Total	
	*Acc*.	*RT*	*Acc*.	*RT*	*Acc*.	*RT*	*Acc*.	*RT*
*Standard (I—C)*	-0.03 (0.15)	56.39 (66.10)	-0.06 (0.18)	49.66 (65.41)	-0.08 (0.15)	100.97 (103.98)	-0.05 (0.15)	67.77 (80.24)
*Reverse (I—C)*	-0.20 (0.22)	74.37 (129.92)	-0.22 (0.15)	62.15 (118.72)	-0.26 (0.32)	-17.15 (73.31)	-0.22 (0.24)	54.02 (81.49)
*Mixed (I—C)*	-0.35 (0.16)	77.57 (85.46)	-0.27 (0.19)	100.68 (82.96)	-0.35 (0.22)	78.35 (75.82)	-0.32 (0.19)	84.42 (80.09)

Response time measured in ms and accuracy measured in percent correct. I = incongruent, C = congruent. SD in parentheses.

In the Reverse Flanker Fish task, a main effect of condition was found for accuracy (F(1,34) = 37.41, p < 0.001, η^2^ = 0.34) and for response time (F(1,34) = 4.53, p < 0.05, η^2^ = 0.02) wherein participants were more accurate and faster in the incongruent trials (Table 4). Again, no significant interaction effects for either with group (*accuracy*: F(2,34) = 0.24, p > 0.05, η^2^ = 0.01); *response time*: F(2, 34) = 2.08, p> 0.05, η^2^ = 0.02).

In the Mixed Flanker Fish task, a main effect of condition was found with both accuracy (F(1,33) = 131.53, p < 0.001, η^2^ = 0.60) and response time (F(1,33) = 13.57, p < 0.001, η^2^ = 0.11), indicating that participants were faster and more accurate on the congruent trials than the incongruent trials within the Mixed task. However, a significant group by condition interaction was not found for either accuracy (F(2,33) = 1.01, p > 0.05, η^2^ = 0.02) nor response time (F(2,33) = 2.32, p > 0.05, η^2^ = 0.04).

#### fMRI Stroop task

**Fig 3:**
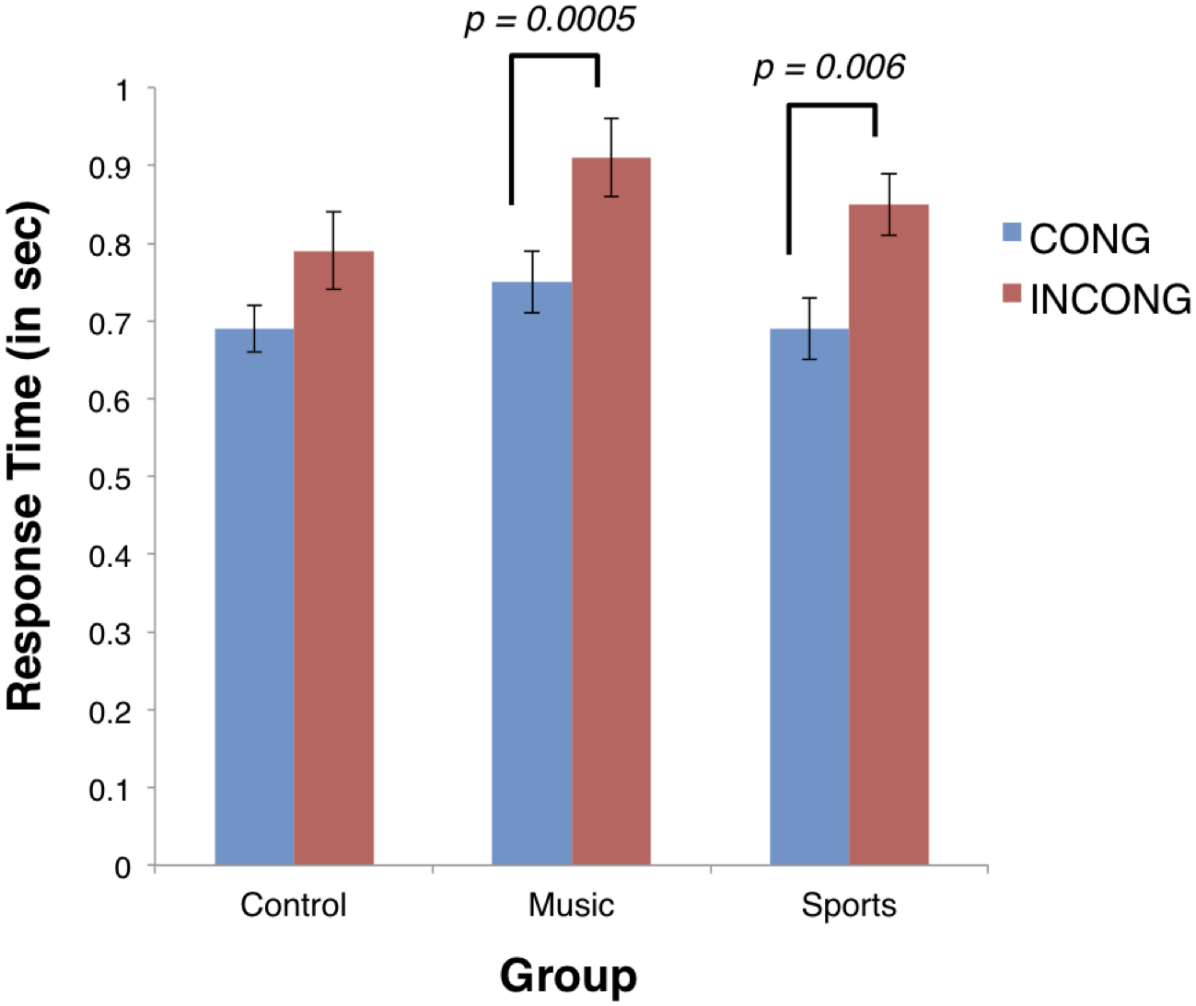
Response times inside the scanner during fMRI Color-Word Stroop task for each condition by group. Response time is measured in seconds. Error bars represent standard error.

For response times during the Color-Word Stroop task inside the scanner, a significant main effect of condition was found (F(1,41) = 26.93, p < 0.001, η^2^ = 0.16), where average response time to congruent trials was faster than response time to incongruent trials. The group by condition interaction was not significant, however (F(2,41) = 0.67, p > 0.05, η^2^ = 0.01). To verify the Stroop effect, we evaluated response times between the two conditions (congruent and incongruent) within each group using paired t-tests. Response times during the incongruent condition were significantly longer in both the music (*incongruent*: M = 910ms, SD = 180ms; *congruent*: M = 740ms, SD = 150ms; t(13) = -4.52, p < 0.001) and sports group (*incongruent*: M = 910ms, SD = 150ms; *congruent*: M = 690ms, SD = 140ms; t(12) = -3.28, p < 0.05), but not the control group (*incongruent*: M = 790ms, SD = 200; *congruent*: M = 690ms, SD = 140ms; t(16) = -2.01, p > 0.05) when correcting for multiple comparisons using a Bonferroni correction (Fig 3, Table 2).

### Whole brain fMRI results

**Fig 4:**
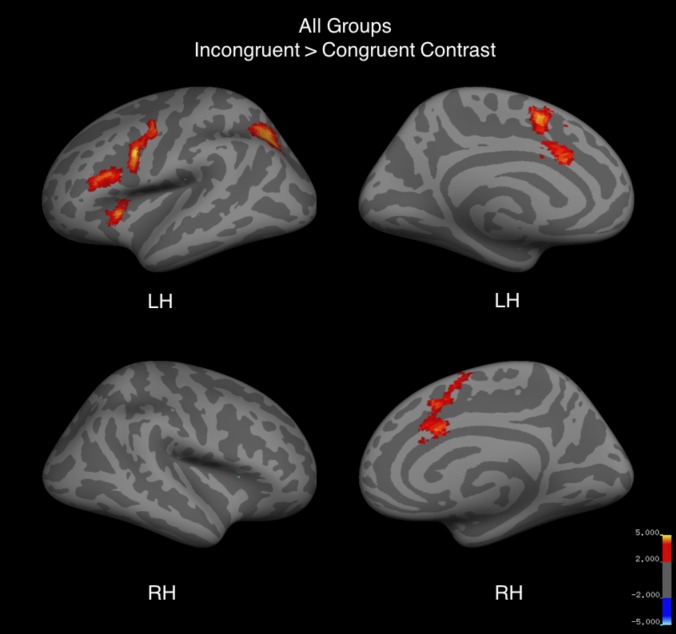
Whole brain activation for incongruent > congruent trials of the fMRI Color-Word Stroop task for all three groups. Red to yellow corresponds to positive z-values. Images are cluster thresholded at Z > 2.3 and cluster size threshold of p < 0.05.

**Fig 5:**
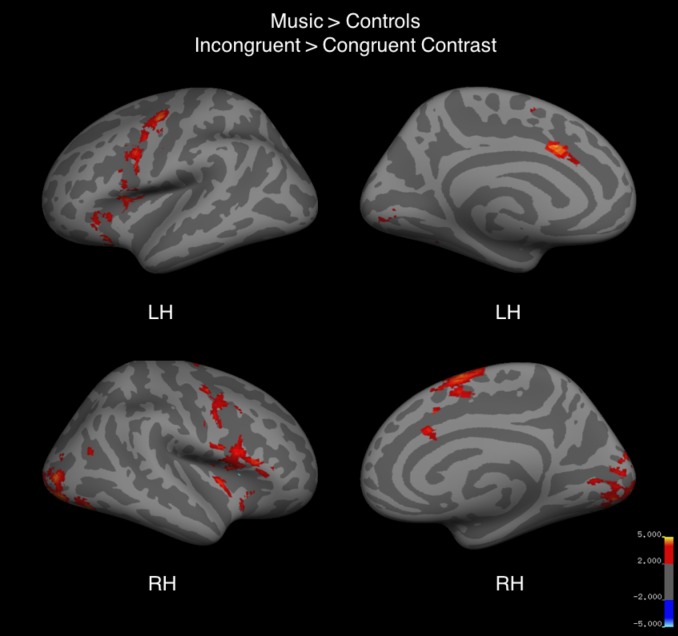
Whole brain activation for incongruent > congruent trials of the fMRI Color-Word Stroop task in a two-sample comparison of music group greater than control group. Red to yellow corresponds to positive z-values. Images are cluster thresholded at Z > 2.3 and cluster size threshold of p < 0.05.

Whole-brain analysis for the contrast incongruent conditions > congruent conditions revealed significant signal differences in all three groups bilaterally in the pre-SMA/SMA and ACC as well as in the left precentral gyrus, anterior insula, IFG, and posterior intraparietal sulcus (Fig 4, Table 5). Musicians, as compared to the control group, showed significantly greater BOLD signal difference between the incongruent and congruent conditions bilaterally in the pre-SMA/SMA, precentral sulcus, insula, ACC, IFG, lateral occipital cortex, and cerebellum (Fig 5, Table 5). The opposite contrast of control group over music group revealed no significant differences in BOLD signal.

**Table 5:** Significant clusters for incongruent > congruent trials of the fMRI Color-Word Stroop task.

Group	z-score	x	y	z	Hemisphere	Region
All	5.34	-2	2	58	left	pre-SMA/SMA
	4.97	2	10	62	right	Pre-SMA/SMA
	4.55	-10	20	28	left	Anterior cingulate
	3.85	6	16	32	right	Anterior cingulate
	4.91	-42	0	32	left	Precentral gyrus
	4.41	-30	20	6	left	Anterior insula
	4.29	-46	30	20	left	Inferior frontal gyrus
	4.4	-24	-62	42	left	Posterior intraparietal sulcus
Music > Controls	4.75	38	-88	-12	right	Lateral occipital cortex
	3.62	-34	-92	-14	left	Lateral occipital cortex
	3.58	24	-64	-22	left	Cerebellum
	2.70	-18	-66	-22	right	Cerebellum
	3.76	12	-86	-10	right	Lingual gyrus
	4.35	-34	20	-14	left	Anterior insula/frontal operculum
	3.74	38	2	6	right	Insula
	3.20	-46	12	20	left	IFG, pars opercularis
	3.92	56	18	24	right	IFG, pars opercularis
	3.79	-42	-2	32	left	Precentral gyrus
	3.85	38	-2	50	right	Precentral gyrus
	4.22	-2	2	58	left	Pre-SMA/SMA
	4.26	2	10	56	right	Pre-SMA/SMA
	4.15	-12	12	34	left	Anterior cingulate
	3.55	8	22	32	right	Anterior cingulate
Music > Sports	3.68	-28	-98	4	left	Lateral occipital cortex

Coordinates represent the peak voxel of the cluster in MNI space.

When comparing the music group with the sports group, no significant differences were found in brain regions that are part of the cognitive control network. The music group did show a significantly greater difference in signal change in the left occipital cortex than the sports group in the incongruent > congruent contrast. The reverse comparison (sports > music) and the comparison of the sports group with the control group revealed no significant voxels.

In the model that included differences in response times during the two conditions of the behavioral Color-Word Stroop task completed outside of the scanner, BOLD signal was not found to be significantly correlated with response time. Adding this covariate to the model did not change the pattern of results regarding the group differences and were therefore not presented.

### Percent signal change in ROI

**Fig 6:**
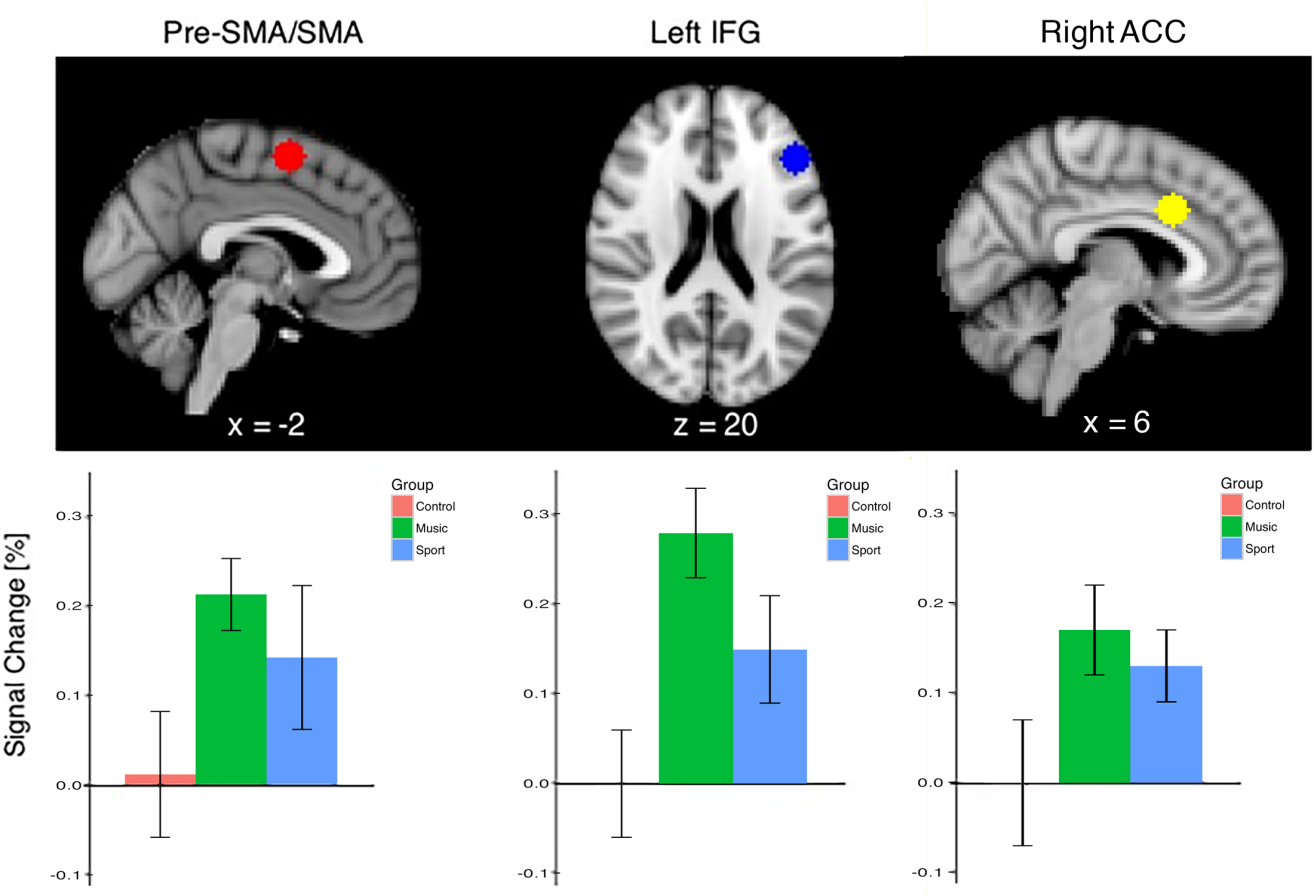
Percent signal change in the pre-SMA/SMA, left IFG and right ACC for the incongruent > congruent contrast during the Color-Word Stroop task by group. Region was defined by the peak voxel within significant clusters. Error bars represent standard error.

The difference in BOLD signal between the music and control group during the Stroop task was further evaluated in an ROI analysis. The percent signal change in regions of interest in the pre-SMA/SMA, left IFG, and left ACC for each contrast revealed that the music group had greater BOLD signal in these regions during incongruent blocks than both the control and sports group (Fig 6).

Given that no differences in behavioral measures of response inhibition were found between the three groups, we attempted to better understand the differences in fMRI by correlating the percent signal change in regions found to be significantly activated by the task with the behavioral measures outside of the scanner. Accuracy and response time during the incongruent trials of the behavioral Color-Word Stroop task, trials in which response conflict was present [64], were correlated with percent signal change in the pre-SMA/SMA, right ACC, and left IFG across all participants. Again, because BOLD signal during the conditions of the Stroop task was correlated with accuracy and response time measured on a separate day, outside of the scanner, we avoid any nonindependency issues [69].

**Fig 7:**
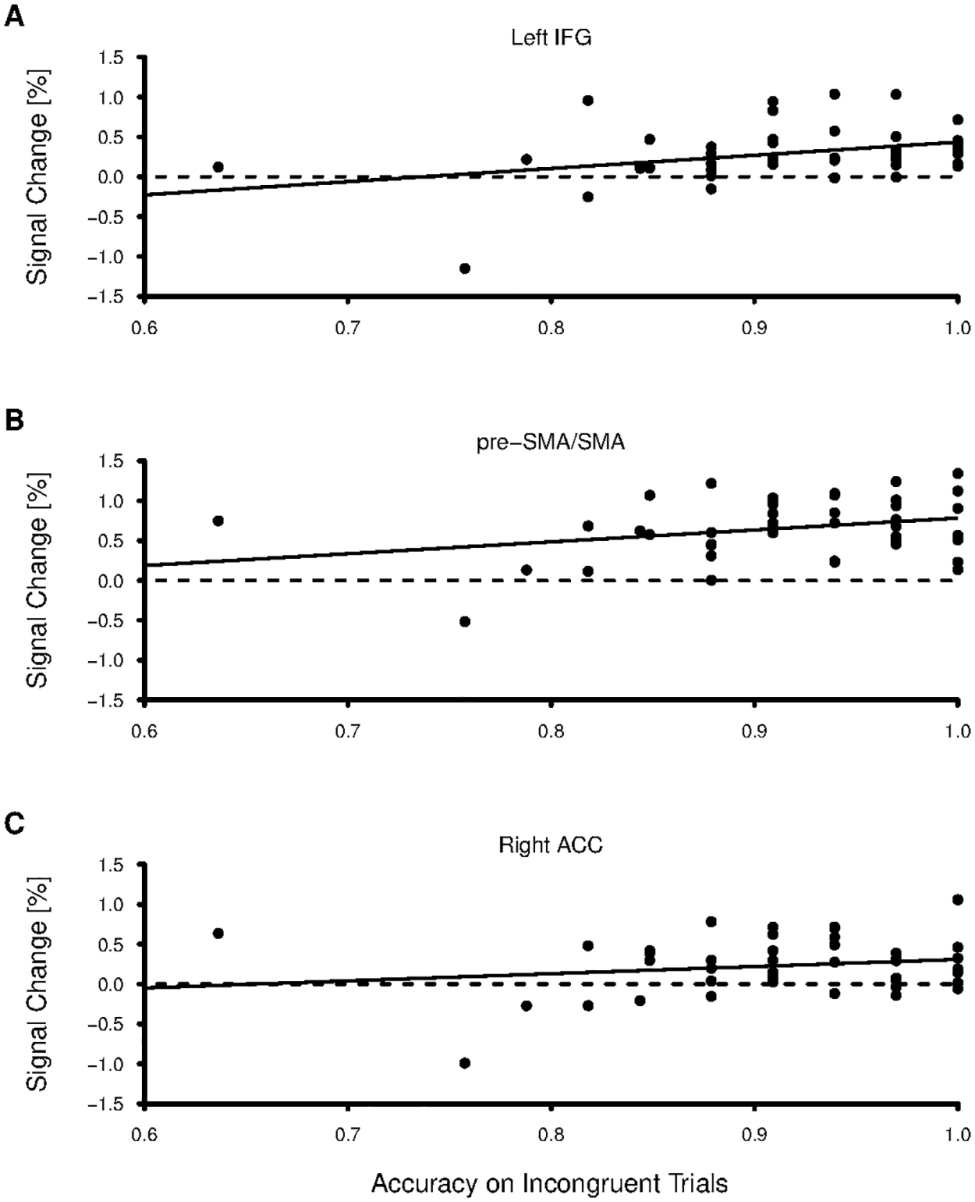
Linear regression plots between accuracy on incongruent trials of the behavioral Color-Word Stroop task and percent signal change in the (A) left IFG, (B) pre-SMA/SMA, and (C) right ACC for the incongruent > congruent contrast during the fMRI Color-Word Stroop task. No correlations were significant after correction for multiple comparisons.

Average accuracy during incongruent trials was positively correlated with percent signal change between incongruent and rest blocks in the left IFG (r = 0.34, p = 0.03) and pre-SMA/SMA (r = 0.30, p = 0.05), but not in the right ACC (r = 0.19, p = 0.22; see Table 6, Fig 7). However, neither of the significant correlations at p < 0.05 survived correcting for multiple comparisons. Furthermore, neither response time during the incongruent trials nor the difference in response time between incongruent and congruent trials were significantly correlated with percent signal change in any of the regions of interest.

**Table 6:** Pearson correlations of percent signal change between conditions of the fMRI Color-Word Stroop task in three regions of interest (pre-SMA/SMA, right ACC, and left IFG) during the and performance on the Behavioral Color-Word Stroop task outside of the scanner.

	Left IFG		Right ACC		Pre-SMA/SMA	
	*I > C*	*I > R*	*I > C*	*I > R*	*I > C*	*I > R*
*Accuracy*	0.31*	0.34*	0.15	0.18	0.19	0.30*
*Response time*	0.09	0.09	0.04	0.18	0.004	0.10

Accuracy refers to the number of correct trials during the incongruent condition. Response time refers to the difference between incongruent and congruent trials. Regions of interest were defined based on peak voxels in the group-level, incongruent > congruent contrast.

*represents significance correlation at p < 0.05 (uncorrected).

## Discussion

The results from our study contribute to the growing body of literature proposing a connection between music training and executive functions. By utilizing several behavioral tasks that disentangle the various components of executive function and two matched control groups that were shown to have equal cognitive abilities prior to onset of training, we were able to evaluate the relationship between early childhood music training and the functioning of brain networks involved in executive functions. Despite the absence of behavioral differences in performance on tasks of response inhibition, working memory, or task switching, we found that children with two years of music training displayed a greater difference in BOLD signal in brain regions involved in conflict processing when compared to a control group that had received no systematic training.

In all participants, incongruent versus congruent trials of the Color-Word Stroop task were associated with activity in a network of brain regions that are known to be involved in cognitive control, including the bilateral pre-SMA/SMA and ACC, as well as the left precentral gyrus, anterior insula, and IFG [70]. Comparing the music group to the control group that did not receive systematic training, we found significantly greater BOLD signal in the music group located bilaterally in the IFG, pre-SMA/SMA, ACC, precentral gyrus, and insula when contrasting incongruent blocks with congruent blocks of the Color-Word Stroop task. Because we did not have a direct measure of performance inside the scanner and behavioral measures on the task did not differ between the three groups, it is difficult to interpret the significance of these findings in terms of the unique connection between music training and executive functions. Considering these limitations, in what follows, we discuss the potential significance of the neural differences between the music and control group in terms of the hypothesized role that each of these regions plays in cognitive control.

The SMA and pre-SMA are structurally and functional connected to the IFG [71,72] and appear necessary for self-initiated actions [73] as well as motor response inhibition [72]. The pre-SMA is consistently found to be more active during incongruent trials of the Stroop task [74]. SMA and pre-SMA activity is observed when comparing participants with shorter reaction times to those with longer-reaction times on a Stop-signal task [75] and patients who have lesions primarily in these areas have impaired performance on similar tasks [76]. Children with ADHD have shown reduced fMRI signal in the pre-SMA during a Go/No-Go task [77,78] as well as decreased cortical thickness in the medial frontal wall that includes the pre-SMA[79], indicating that atypical functioning in the pre-SMA/SMA may be central to deficits in response selection and inhibition seen in certain clinical populations [80].

In our study, the music group showed significantly greater recruitment of both the pre-SMA and SMA during cognitive control. These findings are consistent with previous research that found greater pre-SMA and SMA engagement in both adult and child musicians as compared to controls during a task that probed components of executive function [51,52]. This result may therefore indicate early changes in the cognitive control network in children with several years of music training, particularly in the regions involved in selecting appropriate responses and inhibiting reflexive, inappropriate responses.

The ACC is frequently cited as being involved in cognitive control [81–83], though recently the anatomical and functional distinctions between the ACC and the pre-SMA/SMA have been a topic of debate in the literature (see [84]. In our results, the significant voxels appear on both the dorsal and ventral side of the cingulate sulcus, which is considered the anatomical boundary between the two regions [85]. We therefore labelled the voxels on the ventral side as belonging to the pre-SMA/SMA and voxels on the dorsal side as part of the ACC.

In terms of the specific role of the ACC in cognitive control, the region may be thought of as a conflict monitor: detecting a conflict when it is present and sending a signal to other regions and networks, such as the SMA/pre-SMA, to resolve it [86]. ACC activity was shown to be related to efficient response inhibition only when the trials that required inhibition were frequent, and thus less salient, whereas pre-SMA activity was related to efficient response inhibition regardless of the frequency of conflict trials [87]. Furthermore, activity in the ACC during a Stroop task was positively correlated with age and performance on the task [66] and activity in this region appears to underlie the link between cognitive control abilities and academic success [39]. During an fMRI Color-word Stroop task, we found that children with music training demonstrated increased activity in the ACC as compared to the control children with no music training, which may reflect increased recruitment of the cognitive control network. Speculatively, this may suggest that music training can become linked to the development of various cognitive skills by reinforcing the neural mechanisms in place for saliency detection and conflict monitoring. However, given that this study did not find any behavioral improvement, nor a neural difference between music and sports group, follow-up testing will be needed to validate this link and assess whether music training is unique in its ability to alter the network involved in cognitive conflict.

More recent accounts of ACC functioning emphasize its role in predicting the likelihood of an event or potential outcome and updating the systems for creating expectations in the future [36]. Given that playing a musical instrument involves adjusting responses to match expected outcomes and actions, and the findings that music training might be associated with cross-modal prediction [21], it may be that this increased ACC activity associated with a task of cognitive conflict in the music group is indicative of an enhanced ability to predict conflict likelihood and/or assess the value of exerting control necessary to resolve the conflict at hand.

The IFG, in the right hemisphere in particular, appears to be crucial for inhibitory control and conflict processing [88]. Part of the ventral attention network, the inferior frontal cortex is believed to orient attention towards salient and behaviorally relevant information [89]. Successful stop trials on the stop-signal task, as compared to unsuccessful stop trials, activates the right IFG [90] and patients with lesions in the right IFG displayed worse performance on the task [91]. Moreover, performance on the Go/No-go task is correlated with measures of white matter integrity in the right IFG [92] and children with ADHD [93] and autism [94], two clinical populations with well-documented impairments in cognitive control, displayed hypoactivation in the right IFG during various tasks that require response inhibition. Greater IFG involvement during an inhibition task may therefore reflect additional attentional processing required of conflict trials, ultimately serving to allocate the resources necessary for successful motor inhibition [72].

Furthermore, previous studies have shown involvement of right IFG during control tasks in adults but not children [95] suggesting that right IFG prominence during cognitive control may emerge later in development [96]. Right IFG involvement during the Stroop task was only found in the music > control group-level contrast. We speculate that the increased recruitment of the right IFG observed in the music training group, which was not observed in the control group, could indicate an early shift in the cognitive control neural network from the left to the right IFG, a process that appears to occur naturally with age [90].

BOLD signal in the insula has also been shown to be positively correlated with task performance during cognitive control [97]. On the other hand, several studies have observed greater activity in the insula after unsuccessful trials [98] as well as during trials in which participants took longer to respond [99]. These results suggest that the insula may not be directly involved in the inhibition of a motor response, but rather reflect a participant’s level of motivation or focus during the task [97]. In this view, the insula generates the negative affective response associated with making a mistake, which, in turn, leads to added incentive to perform well in future trials [100]. This is in line with the insula’s well-documented role more generally in detecting salient events and initiating the appropriate behavioral responses [101], as well as in feelings of uncertainty that stem from decision-making [102]. The significant difference of BOLD signal in the insula observed in the music group, but not the control group, might therefore indicate that early childhood training is associated with improved monitoring of emotional responses during conflict processing, which fosters more successful actions and decisions in the future.

### Limitations

Although we are interpreting the fMRI findings as evidence for more advanced and effective conflict processing in the music group compared to the control group, we recognize that some investigators have argued that increased activation of cortico-striatal regions during control or inhibition tasks is indicative of greater cognitive conflict [103]. BOLD signal in the left IFG has been found to be is greater in older adults who perform more poorly on the Stroop task [104]. Moreover, ACC activity has been observed during tasks that require higher cognitive load [105]. Given these results, it may be that the greater activation in these regions observed in the music group reflects increased effort required to resolve the conflict, whereas decreased activation reflects more efficient invocation of the networks needed to implement cognitive control [106]. Without a measure of task performance inside the scanner, we recognize that we cannot be certain that our findings do not indicate lower efficiency in cognitive control regions in the music group. However, given that the percent signal change in the pre-SMA/SMA and left IFG during the response inhibition trended towards being significantly correlated with higher accuracy on incongruent trials of the behavioral Stroop task, we reason that the greater difference in activity in cortico-striatal structures and the recruitment of the right IFG observed in the music group more likely reflect improvements in cognitive control.

An alternative hypothesis that was not addressed in this study, is the role of motivation on executive functioning tasks. Previous findings suggest that positive motivation enhances cognitive control [107] and that activity in the medial and lateral frontal cortex during cognitive control tasks were greater for high versus low reward conditions [108]. Though response time during the Stroop task did not significantly differ by group, the fact that the music group still demonstrated longer response times on the task could be reflective of increased motivation or self-discipline to perform the task correctly. Furthermore, in this study, we do not have a direct measure of how motivated the children were to participate in their respective extracurricular activities and we therefore cannot rule out the possibility that the neural differences found could pertain to increased engagement with and commitment to the training programs, rather than a unique property of the training itself. We hope to include a measure of motivation in subsequent follow-up assessments.

We chose a block design for the fMRI portion of this study to simplify the task for our participants, which we acknowledge has certain limitations. Other researchers have suggested that an event-related design is more appropriate with the Stroop task inside the scanner because it allows for intermixing conditions and is less susceptible to habituation to the task, resulting in more robust activation maps associated with the Stroop-effect [67]. However, similar Stroop effects have been reported using block-designs [67]. Because of this, and given the age of our participants at baseline, we decided to use the easier task that does not require task-switching inside the scanner. Future studies with a different cohort of children may be able to use an event-related design with more extensive pre-training.

Despite finding differences in BOLD signal between the music group and control group, no differences were found in either response time during the fMRI task or in accuracy/response time during the behavioral executive function tasks outside of the scanner. We are not the first to report differences between groups in neural pathways involved in executive function in the absence of behavioral differences [51,94,109,110]. As other researchers have proposed, differences in brain responses in the absence of behavioral differences could be the result of varying cognitive strategies used to complete the task [111]. Alternatively, the lack of behavioral findings could be due to the low statistical power stemming from the relatively small number of participants in each group and the relatively low amount of trials in each task. We additionally had to remove data from several participants based on poor performance during the task, which can commonly occur due to fatigue, boredom, and/or difficulty in understanding and following instructions. We recognize the drawbacks of conducting a study aimed at assessing three groups of developing children on a wide range of tasks that probe both cognitive and socio-emotional development, such as limited sample size, limited time dedicated to each task, and difficulties with task compliance. That being said, while the lack of group differences on performance during the Stroop task both inside and outside of the scanner makes an interpretation of the fMRI findings more difficult, based on the findings from previous neuroimaging studies on the development of executive function as well as our own findings of a positive correlation between signal change in both the pre-SMA/SMA and left IFG and performance on the behavioral Stroop task, we speculate that our neuroimaging result suggest early changes in pathways involved in executive function that behavioral measures of inhibitory control are not sensitive enough to detect.

Furthermore, no significant neural or behavioral differences were found between the music group and the sports group, suggesting that any type of training in which a child focuses on developing a particular skill through repeated practice may be associated with change in the neural organization of networks involved in cognitive control. Playing a sport as well as a musical instrument both require cognitive planning, switching attention between various tasks, keeping information in working memory, and executing and inhibiting fine motor actions [112]. Other mechanisms could additionally account for far-transfer effects of both types of training, such as the self-discipline and self-motivation that is required to learn a new skill, or the social interactions that come with working together to achieve some larger goal. However, we are cautious to interpret these null findings as evidence for far-transfer effects associated with sports training. Given that no differences were found between the sports group and control group either, it may be that music training does influence conflict processing more robustly than sports training, but, because children in all groups were developing naturally with age, differences are only detectable on the extreme ends of the spectrum and the sports group fell somewhere in the middle. Still, it is quite possible that neural differences do exist between the music and sports group that are not detectable here due to the small sample size of each group and low statistical power. Given these limitations, it remains difficult to conclude that music training impacts the development of executive functions more so than other types of engaging and demanding activities in childhood.

### Conclusions

To summarize, after two years of community, group-based music training, children showed significantly greater differences in the cognitive control network during the Color-Word Stroop task as compared to a control group that was not involved in any systematic afterschool activities. Performance on several behavioral tasks of executive function did not vary by group, though there is modest evidence that the degree of activation of these regions increased with better performance on a behavioral Stroop task. The results from this study provide support for the hypothesis that learning to play a musical instrument can impact brain networks that enable executive functioning, which, in turn, may mediate the link between music training and enhanced cognitive abilities [51]. Given that we did not find any differences in behavioral measures of executive functioning, it may be that functional imaging is uncovering early development of neural systems that have not yet manifested into measurable changes on behavioral tasks. In addition, no significant differences in BOLD signal during the Stroop task were found between the music group and a group of children that received an equal amount of sports training nor between the sports and control group. Therefore, while music training may indeed influence the development of neural systems involved in cognitive control, the results from this study cannot rule out the possibility that other types of focused, challenging, and repeated training may do so as well. Continued examination of the same participant population over the next few years will hopefully yield answers to these outstanding questions.
